# The Biosynthesis, Signaling, and Neurological Functions of Bile Acids

**DOI:** 10.3390/biom9060232

**Published:** 2019-06-15

**Authors:** Yoshimitsu Kiriyama, Hiromi Nochi

**Affiliations:** Kagawa School of Pharmaceutical Sciences, Tokushima Bunri University, Shido 1314-1, Kagawa, Sanuki 769-2193, Japan; kiriyamay@kph.bunri-u.ac.jp

**Keywords:** bile acids, FXR, TGR5, SHP, Alzheimer’s disease, Parkinson’s disease, Huntington’s disease, ALS

## Abstract

Bile acids (BA) are amphipathic steroid acids synthesized from cholesterol in the liver. They act as detergents to expedite the digestion and absorption of dietary lipids and lipophilic vitamins. BA are also considered to be signaling molecules, being ligands of nuclear and cell-surface receptors, including farnesoid X receptor and Takeda G-protein receptor 5. Moreover, BA also activate ion channels, including the bile acid-sensitive ion channel and epithelial Na^+^ channel. BA regulate glucose and lipid metabolism by activating these receptors in peripheral tissues, such as the liver and brown and white adipose tissue. Recently, 20 different BA have been identified in the central nervous system. Furthermore, BA affect the function of neurotransmitter receptors, such as the muscarinic acetylcholine receptor and γ-aminobutyric acid receptor. BA are also known to be protective against neurodegeneration. Here, we review recent findings regarding the biosynthesis, signaling, and neurological functions of BA.

## 1. Introduction

Bile acids (BA) are synthesized from cholesterol in the liver and are a significant component of bile. They are stored in the gallbladder and released into the small intestine after a meal. BA are amphipathic steroid acids and are known as indispensable detergents, expediting the digestion and absorption of dietary lipids and lipophilic vitamins by forming micelles in the small intestine. Recently, they have also been considered to be signaling molecules. They are now recognized as ligands of farnesoid X receptor (FXR), a nuclear hormone receptor, and Takeda G-protein receptor 5 (TGR5), a G-protein-coupled receptor (GPCR) that is also known as G protein-coupled bile acid receptor 1 (GPBAR1) [1].

Bile salt composition of a variety of vertebrate species is validated. Bile salts include C_27_ bile alcohols, C_27_ bile acids, and C_24_ bile acids. C_24_ bile acids exist in all vertebrates, although C_27_ bile alcohols and C_27_ bile acids exist in fish, amphibians, reptiles, and birds [2]. 5α-C_27_ bile alcohol sulfates are considered as the first ancestral bile salts. 5α-cyprinol sulfate is 5α-C_27_ bile alcohol sulfate and activates FXR from the frog and the zebrafish, but not from the human and the mouse. In contrast, taurochenodeoxycholic acid (TCDCA) and lithocholic acid (LCA) activate FXR from the human and the mouse, but not from the frog and the zebrafish. Thus, the structure of FXR may be changed to adapt to bind species-specific bile salts [3].

BA regulate glucose and lipid metabolism by activating these receptors in various peripheral tissues, including the liver, brown and white adipose tissue, skeletal muscle, and the pancreas [4]. In addition, a recent study has shown that 20 different kinds of BA can be found in the central nervous system (CNS) and the presence of two primary bile acids, chenodeoxycholic acid (CDCA) and cholic acid (CA), in the brain is essential [5,6]. In this review, we focus on recent advances in the understanding of the biosynthesis, signaling, and neurological functions of BA.

## 2. Synthesis of BA

**Figure 1:**
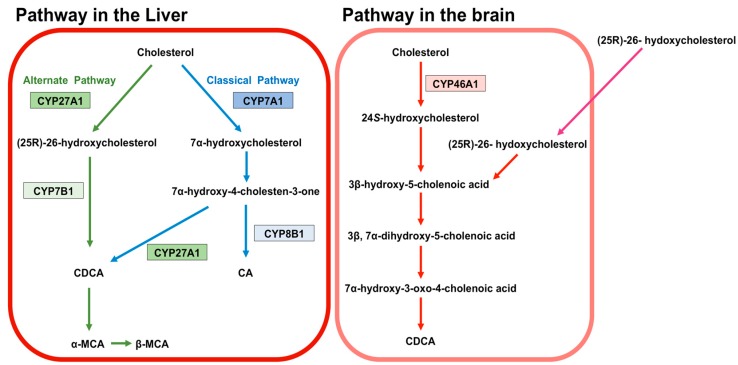
Synthesis of bile acids (BA). The classical pathway is initiated by cytochrome P450 7A1 (CYP7A1). CYP7A1 converts cholesterol to 7α-hydroxycholesterol. 7α-hydroxycholesterol is then converted to 7α-hydroxy-4-cholesten-3-one. Cytochrome P450 8B1 (CYP8B1) leads the production of CA from 7α-hydroxy-4-cholesten-3-one. 7α-hydroxy-4-cholesten-3-one is also converted to CDCA by cytochrome P450 27A1 (CYP27A1). The alternative pathway begins with converting cholesterol to (25R)-26-hydroxycholesterol by CYP27A1. Cytochrome P450 7B1 (CYP7B1) leads (25R)-26-hydroxycholesterol to CDCA. CDCA is converted to α-muricholic acid (MCA), and β-MCA. These BA are then conjugated with glycine or taurine. BA in rodents are also conjugated with taurine in the liver. BA synthesized in the liver are called primary BA. In the brain, 24*S*-hydroxycholesterol is converted from cholesterol by cytochrome P450 46A1 (CYP46A1). 24*S*-hydroxycholesterol is a precursor of 3β-hydroxy-5-cholenoic acid, which can be converted to CDCA through the intermediates (3β, 7α-dihydroxy-5-cholenoic acid and 7α-hydroxy-3-oxo-4-cholenoic acid). A large amount of (25R)-26-hydoxycholesterol incorporates to brain from circulation, and (25R)-26-hydoxycholesterol can also be converted to 3β-hydroxy-5-cholenoic acid.

**Figure 2:**
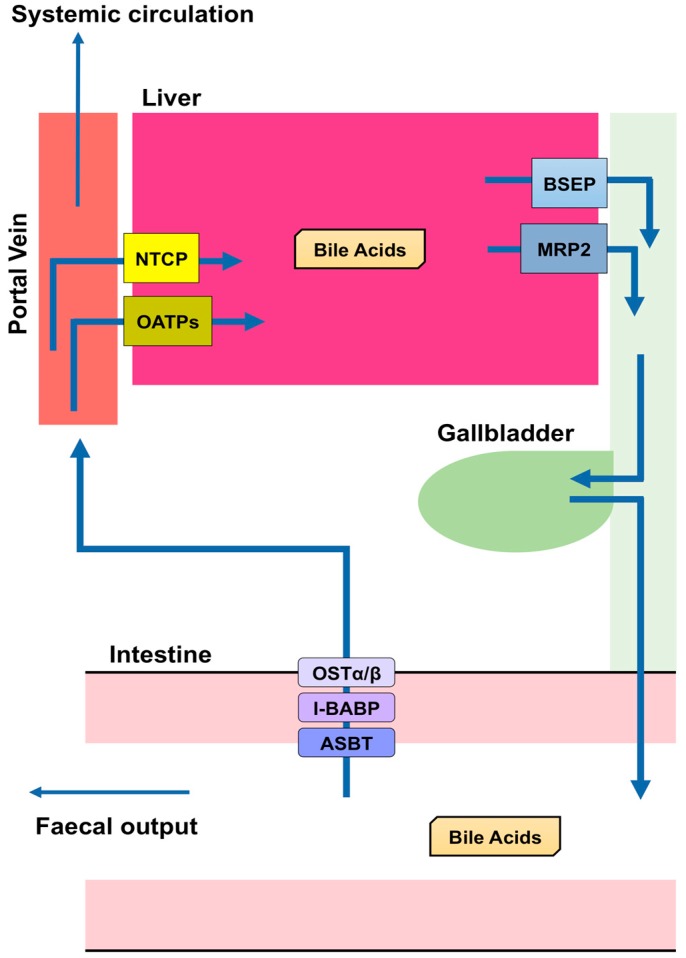
Enterohepatic circulation. Bile salt export pump (BSEP) and multidrug resistance-associated protein 2 (MRP2) are transporters mediating the secretion of bile acids (BA) from hepatocytes to the bile canaliculus in the liver. BA are stored in the gallbladder and secreted into the small intestine after a meal. Most BA (approximately 95%) are reabsorbed, whereas the remainder is excreted with feces. Apical sodium dependent bile acid transporter (ASBT) in the apical brush border of enterocytes takes BA into enterocytes. Ileal bile acid-binding protein (I-BABP) is related to the intracellular transport in enterocytes. The passage of BA through the basolateral membrane of enterocytes into the portal blood occurs through organic solute transporter (OST) α and β. BA released into the blood from the small intestine are transported into the liver by Na^+^-taurocholate co-transporting polypeptide (NTCP) or organic anion-transporting polypeptides (OATPs). These BA are then reconjugated and secreted with newly produced BA. This recycling system is termed enterohepatic circulation.

BA are predominantly produced in the liver, via two biosynthetic pathways: The classical (or neutral) pathway and the alternative (or acidic) pathway [7] (Figure 1). The synthesis of BA from cholesterol involves at least 16 enzymes [8]. The classical pathway is initiated by cytochrome P450 7A1 (CYP7A1), also known as cholesterol 7α hydroxylase. Cholesterol is converted to 7α-hydroxycholesterol by CYP7A1, which is the rate-limiting enzyme in this pathway. 7α-hydroxycholesterol is then converted to 7α-Hydroxy-4-cholesten-3-one. Cytochrome P450 8B1 (CYP8B1), also known as sterol 12α-hydroxylase, is responsible for the production of CA from 7α-hydroxy-4-cholesten-3-one, but 7α-hydroxy-4-cholesten-3-one is also converted to CDCA by cytochrome P450 27A1 (CYP27A1), also known as sterol 27-hydroxylase. The alternative pathway begins with the conversion of cholesterol to (25R)-26-hydroxycholesterol [9] by CYP27A1. Cytochrome P450 7B1 (CYP7B1), also known as oxysterol 7-α-hydroxylase, leads (25R)-26-hydroxycholesterol to CDCA. CDCA is converted to α-muricholic acid (MCA), and β-MCA. These BA are then conjugated with glycine or taurine. BA synthesized in the liver are referred to as “primary BA” [10,11,12]. Primary BA in rodents are also conjugated with taurine in the liver [13]. Bile salt export pump (BSEP) and multidrug resistance-associated protein 2 (MRP2) are the transporters that mediate the secretion of BA from hepatocytes into hepatic bile canaliculi (Figure 2). BSEP is the main transporter of BA, whereas MRP2 principally transports BA glucuronides and sulfates [1,14].

After primary BA are transported to and stored in the gallbladder, they are secreted into the small intestine, where the intestinal microbiota converts them into secondary BA. Conjugated primary BA are deconjugated by bacterial bile salt hydrolase [15,16]. In humans, 7α-dehydroxylase converts CA and CDCA to deoxycholic acid (DCA) and lithocholic acid (LCA), respectively [1,16]. CDCA is also converted to ursodeoxycholic acid (UDCA) by 7α-hydroxysteroid dehydrogenase (7α-HSDH) and 7β-HSDH [17]. In rodents, β-MCA is transformed into ω-MCA, which is then metabolized to hyodeoxycholic acid (HDCA), while α-MCA is converted to murideoxycholic acid [18,19].

Most BA molecules (approximately 95%) are reabsorbed, and the remainders are excreted in the feces [20]. This reabsorption largely takes place in the small intestine, but a small proportion also occurs in the colon. Apical sodium dependent bile acid transporter (ASBT) in the apical brush border of enterocytes transports BA into enterocytes [21,22,23,24,25], and a mutation in this transporter causes primary BA malabsorption [22]. Although diabetes is a multifactorial disease, appropriate glycemic control is crucial for the prevention of diabetic complications [26,27,28,29]. Inhibition of the reabsorption of BA by an ASBT inhibitor (GSK2330672) or a BA sequestrant (colesevelam or colestimide) causes a reduction in circulating BA concentrations, which leads to improvements in glycemic control and insulin sensitivity in diabetic patients [30,31]. Ileal bile acid-binding protein (I-BABP), also known as fatty acid binding protein 6, mediates intracellular transport in the enterocyte [21,32], and the transport of BA through the basolateral membrane of enterocytes into the portal circulation is performed by organic solute transporter (OST) α and β. OSTα and OSTβ are localized to the basolateral membranes of the enterocyte [21] and expressed as heterodimers or heteromultimers [33,34,35]. BA released into the blood from the small intestine are transported to the liver, reconjugated, and secreted with newly synthesized BA. Thus, BA secreted from the liver into the intestine are recycled, and this recycling system is referred to as the “enterohepatic circulation” (Figure 2).

The transport of BA from the portal circulation into hepatocytes is performed by Na^+^-dependent and Na^+^-independent pathways. Na^+^-dependent transport of BA requires Na^+^-taurocholate co-transporting polypeptide (NTCP), while Na^+^-independent transport is executed by organic anion-transporting polypeptides (OATPs). NTCP is localized to the basolateral membrane of hepatocytes and is the main transporter responsible for transporting BA into hepatocytes using a Na^+^-dependent mechanism [36,37,38]. OATP1B1 and OATP1B3 are members of the OATP family that are expressed in the basolateral membrane of hepatocytes [39]. Oatp1b2 is the rodent ortholog of human OATP1B1/3 [40]. BA conjugated with glycine or taurine are more effectively transported into cells by OATP1B1/3 than unconjugated BA [41]. However, BA in the portal vein are not completely reabsorbed by hepatocytes (Figure 2): The concentration of BA in the portal vein is 10–80 μM and that in the systemic circulation of humans and rodents is ∼2–10 μM [10].

## 3. BA in the Brain

Both conjugated and unconjugated BA can be detected in the brain of humans and rodents [5,6,42]. Twenty BA have been identified in the rat brain, consisting of nine unconjugated BA and eleven conjugated BA [6]. Most of these 20 BA are also found in the blood of rats [43]. CDCA is present in the highest concentration in the rat brain [5]. Although the origin of brain BA remains unclear, they can be synthesized in the brain or transported into the brain from the peripheral circulation by BA transporters and/or diffuse across the blood-brain barrier (BBB). Conjugated BA require transporters to cross the BBB, because they are large and negatively-charged at physiological pH [44,45], and indeed, transporters for both the uptake and efflux of BA, such as NTCP, OATP1, BSEP, and MRP2, have been identified in the choroid plexus and brain capillaries [46,47,48]. In addition, BA in the bloodstream can themselves induce permeability of the BBB. The BBB is formed by brain microvascular endothelial cells that are bound to adjacent cells via tight junctions [49]. Occludin is one of the membrane proteins that are responsible for tight junction formation in the BBB [50], and its phosphorylation by BA via ras-related C3 botulinum toxin substrate 1 (RAC1) increases the permeability of the BBB [51]. In contrast, unconjugated BA, such as CA, CDCA, and DCA, are lipophilic, even though they are ionized [52]. Therefore, unconjugated BA might be able to cross the BBB by passive diffusion, and indeed, there are strong correlations between the concentrations of unconjugated BA (CA, CDCA, and DCA) in the brain and the serum [53,54].

24*S*-hydroxycholesterol predominantly exists in the brain of humans and rodents and is generated from cholesterol in the brain by cytochrome P450 46A1 (CYP46A1), also known as cholesterol 24-hydroxylase [55,56,57,58] (Figure 1). 24*S*-hydroxycholesterol is a precursor of 3β-hydroxy-5-cholenoic acid, which can be converted to CDCA through the intermediates (3β, 7α-dihydroxy-5-cholenoic acid and 7α-hydroxy-3-oxo-4-cholenoic acid) with rat brain extracts, although this CDCA-generating activity is very low [59]. Moreover, a large amount of (25R)-26-hydoxycholesterol incorporates to brain from circulation, and (25R)-26-hydoxycholesterol can also be converted to 3β-hydroxy-5-cholenoic acid [60]. Therefore, it is thought that the principal source of BA in the brain is the peripheral circulation.

## 4. Signaling Induced by BA

**Figure 3:**
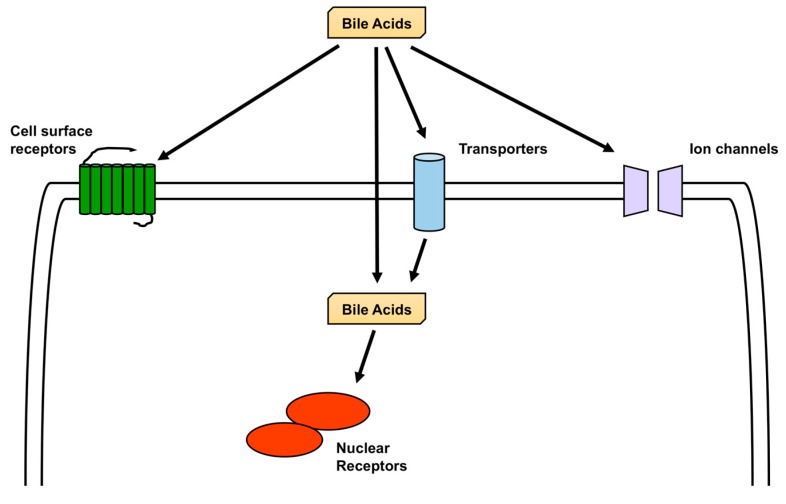
Bile acids (BA) activate receptors and ion channels. Nuclear receptors activated by BA include the farnesoid X receptor (FXR), pregnane X receptor (PXR), vitamin D receptor (VDR), liver X receptor (LXR), and glucocorticoid receptor (GR). Unconjugated BA might be able to cross the plasma membrane, and conjugated BA might cross the plasma membrane using transporters. Cell surface receptors activated by BA are Takeda G-protein receptor 5 (TGR5), sphingosine-1-phosphate receptor 2 (S1PR2), M2 and M3 muscarinic receptors, and formyl-peptide receptor (FPR). Bile acid-sensitive ion channel (BASIC), epithelial Na^+^ channel (ENaC), and large-conductance Ca^2+^- and voltage-activated K^+^ (BK) channels are ion channels that are activated by BA. These receptors and ion channels are expressed in the brain.

FXR and TGR5 are the most studied receptors activated by BA [1], but other nuclear receptors are also activated by BA, including pregnane X receptor (PXR), vitamin D receptor (VDR), liver X receptor (LXR), and glucocorticoid receptor (GR) [61,62], and in addition to TGR5, sphingosine-1-phosphate receptor 2 (S1PR2), M2 and M3 muscarinic receptors, and formyl-peptide receptor (FPR) are cell-surface receptors activated by BA. All these receptors are expressed in the brain [63,64,65,66,67,68]. Furthermore, the bile acid-sensitive ion channel (BASIC), epithelial Na^+^ channel (ENaC), and large-conductance Ca^2+^-and voltage-activated K^+^ (BK) channels are ion channels that are activated by BA and are also expressed in the brain [69,70,71,72,73] (Figure 3).

### 4.1. Activation of Nuclear Receptors by BA

FXR is a nuclear hormone receptor for which BA are ligands [74,75,76]. Unconjugated BA, such as CDCA, DCA, and LCA, are high-affinity ligands of FXR [74,75,76], and CA and conjugated BA can effectively activate FXR in the presence of a BA transporter (NTCP) in the cell [74]. However, taurine-β-MCA (TβMCA), glycine-β-MCA (GβMCA), and DCA act as antagonists at FXR inhibiting FXR signaling [77,78,79]. FXR monomers and heterodimers with a retinoid-X receptor (RXR) bind to their target sequence on DNA molecules, which is an inverted repeat of AGGTCA separated by one nucleotide (IR1) [80,81]. Moreover, FXR can also bind to IR0, an everted repeat of AGGTCA separated by two or eight nucleotides (ER2 or 8), and a direct repeat of AGGTCA separated by one, four, or five nucleotides (DR1, 4, or 5) [80,82,83,84]. Comparison of the available DNA binding sites for FXR in the liver and the intestine has shown that they only have 11% in common [80], suggesting that the pattern of gene expression induced by FXR is tissue-specific, and, thus, that the genes regulated by FXR in the brain might be different from those in peripheral tissues. 

PXR, VDR, and LXR also heterodimerize with RXR [85]. PXR is also known as the steroid and xenobiotic receptor (SXR) and is activated by many xenobiotics and endobiotics, but also by LCA [86,87,88]. PXR plays an important role in the regulation of the expression of genes encoding transporters of drugs and enzymes, which are involved in drug metabolism. Drug transporters upregulated by PXR include MRP2 and OATP2, and drug-metabolizing enzymes upregulated by PXR include CYP3A and CYP2B, which metabolize LCA to hyocholic acid and UDCA [84,89,90,91,92]. In contrast, CYP7A1 expression is suppressed by PXR, but PXR does not directly bind to the promoter region of the CYP7A1 gene. Instead, PXR suppresses the activation of hepatocyte nuclear factor 4α, which binds to the promoter region of the CYP7A1 gene and upregulates its expression [93,94]. Thus, PXR regulates genes involved in BA synthesis and metabolism. In addition, PXR expression can be upregulated by FXR [95].

VDR is a nuclear receptor that is crucial for the control of calcium homeostasis. Its main endogenous ligand is 1,25-dihydroxyvitamin D3, but LCA and its metabolite, 3-keto LCA, are also able to activate VDR [96,97]. The activation of VDR leads to the upregulation of CYP family proteins and BA transporters, and in particular, LCA can induce CYP3A and MRP3 expression by acting as a VDR ligand [97,98,99,100].

LXRα and β control cholesterol homeostasis and are activated by oxysterols, which are endogenously produced cholesterol metabolites, but HDCA is also a weak activator of LXRα [101,102]. LXRα knockout mice exhibit a reduction in BA pool size and excretion, because LXRα upregulates the expression of CYP7A, which is the key enzyme required for the conversion of cholesterols to BA. In contrast, LXRβ does not affect the expression of CYP7A [103,104,105].

GR is activated by glucocorticoids and induces or represses the expression of large numbers of genes, thereby influencing various physiologic functions, including metabolic homeostasis, stress, inflammation, and development [106]. UDCA, taurocholic acid (TCA), and glycochenodeoxycholic acid (GCDA) induce GR activity [107,108], and the activation of GR by UDCA suppresses nuclear factor-κB activation [108]. Furthermore, the activation of GR by TCA or GCDA reduces the expression of corticotropin-releasing hormone in the hypothalamus [107].

SHP is a nuclear hormone receptor that is induced by the activation of FXR [109] Although it has a ligand-binding domain (LBD), it lacks a DNA binding domain. The endogenous ligand of SHP has yet to be identified. The function of SHP is to repress many nuclear receptors, including PXR, CAR, LXR, GR, and FXR, which is achieved by the LBD of SHP interacting with the LBD of other nuclear receptors to inhibit their transcriptional activity [110]. Therefore, FXR can indirectly repress the expression of a range of genes by inducing SHP.

### 4.2. Activation of Cell-Surface Receptors by BA

TGR5, also known as GPBAR1 or membrane-type receptor for BA (M-BAR), has been identified as a GPCR for BA by two independent groups [111,112]. TGR5 can be activated by LCA, DCA, CDCA, CA, and taurine or glycine-conjugated forms of these BA. LCA and DCA, which are secondary BA, and conjugated forms of LCA and DCA, are the most potent activators of TGR5 [111], but HDCA and UDCA are also weak activators of TGR5 [101,112,113]. TGR5 is expressed in various tissues, including the small intestine, liver, adipose, spleen, brain, and spinal cord. TGR5 activation is involved with the control of glucose metabolism, neuronal function, and the immune system [63], and its activation by BA regulates a variety of signaling intermediates, including protein kinase A (PKA), Akt/PKB (protein kinase B), and mitogen-activated protein kinases (MAPKs) [114]. Because TGR5 is a Gα_s_-coupled GPCR, the activation of TGR5 stimulates adenylyl cyclase to increase the concentration of cyclic AMP (cAMP), activating PKA and exchange proteins directly activated by cAMP (EPACs) [115]. The activation of TGR5 by BA can also activate both PKA and EPACs [116]. PKA and EPACs activate various signaling pathways, including Akt/PKB and MAPKs [117,118,119]. Moreover, TGR5 can also associate with Gα_i_ and Gα_q_, although TGR5 has been identified to be a Gα_s_-coupled GPCR [120,121,122]. In ciliated cholangiocytes, TGR5 is coupled to Gα_i_ and the activation of TGR5 results in a decrease in the concentration of cAMP and the activation of extracellular signal-regulated kinases (ERKs). In contrast, TGR5 is coupled to Gα_s_ and the activation of TGR5 results in an increase in the concentration of cAMP and the suppression of ERK activation in non-ciliated cholangiocytes [121]. Thus, the signaling pathways influenced by the binding of BA to TGR5 vary according to cell type and cellular conditions. In addition, a recent report has shown that the expression of TGR5 can be induced by FXR in the ileum [123].

S1PR2 is one of five subtypes of S1PR (S1PR1-5). S1P is the ligand for the S1PRs and is generated by the phosphorylation of sphingosine by sphingosine kinase 1 or 2 [67,124], but BA can also act as ligands for S1PR2. S1PRs are GPCRs, and S1PR2 can couple with Gα_i_, Gα_q_, or Gα12/13 [67]. S1PR2 is expressed in a variety of tissues and its activation influences various signaling molecules, including adenylyl cyclase, Akt/PKB, and MAPKs [65,67,124]. An unconjugated BA (DCA) and conjugated BA [TCA, TDCA, tauroursodeoxycholic acid (TUDCA), glycocholic acid (GCA), glycodeoxycholic acid (GDCA), and glycochenodeoxycholic acid (GCDC)] are ligands for S1PR2, binding of which leads to the activation of ERKs and Akt/PKB [125,126,127].

M2 and M3 muscarinic receptors are GPCRs that are widely distributed and responsible for many of the physiological effects of acetylcholine. They exist in five subtypes (M_1_–M_5_), of which the M_1_, M_3_, and M_5_ receptors couple to Gα_q_, while the M_2_ and M_4_ receptors couple to Gα_i_ [128]. It has been reported that conjugated BA (TCA, GDCA, and TDCA) are partial agonists of the M_2_ receptor and activate the Gα_i_ pathway [129,130], whereas DCA induces the expression of ALDH1, CD166, and Myc via the M_3_ receptor [131]. TDCA stimulates the relaxation of phenylephrinein-induced constriction in aortic rings, and this TCDCA-stimulated-relaxation is diminished in aortic rings from M_3_ receptor-knockout mice [132].

FPR is also a GPCR and has various ligands, including bacterially and mitochondrially-derived formyl peptides [68]. FPR couples to Gα_i_ and its activation leads to an increase in the mobilization of cellular calcium and the activation of signaling molecules, including MAPKs and Akt/PKB [66,133]. DCA and CDCA also bind to FPR and act as antagonists, suppressing the activation of FPR by formyl N-formyl-met-leu-phe (fMLP), a bacterially-derived FPR ligand [134,135].

### 4.3. Activation of Ion Channels by BA

BASIC is also known as acid-sensing ion channel subunit family member 5 (ASIC5) or intestine Na^+^ channel (INAC) in humans and brain-liver-intestine Na^+^ channel (BLINaC) in rodents. BASIC is a member of the degenerin (DEG)/ENaC ion channel family [72]. CA, UDCA, HDCA, LCA, DCA, and CDCA can activate rodent BASIC. In contrast, only DCA and CDCA can activate human BASIC [136].

ENaC is also a member of the DEG/ENaC ion channel family and consists of α, β, and γ subunits [70,71,137]. In humans, the δ subunit of ENaC is highly expressed in the brain, and δβγENaC also exists in this tissue [71,137]. CA, DCA, CDCA, and their taurine conjugates can activate both δβγENaC and αβγENaC [137].

BK channels are composed of α and β subunits, and there are four isotypes of the β subunit that can be used. LCA, DCA, and CA activate BK channels, and LCA is the most effective BA in this regard [138]. LCA activates the BK channel by direct interaction with the transmembrane domain of the β1 subunit [139]. The β4 subunit is the most abundant subunit in the brain, but the β1 subunit is also expressed in this tissue [73].

## 5. Neurological Functions of BA

**Figure 4:**
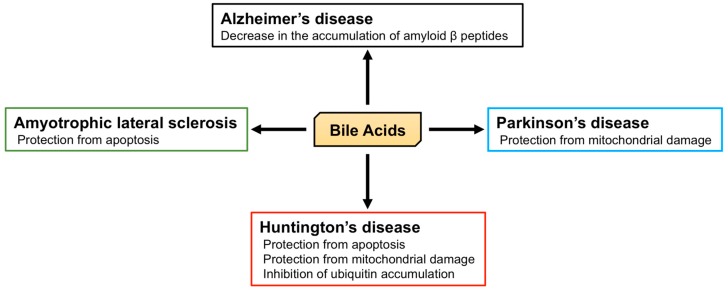
Protective functions of bile acids (BA) against neurodegeneration. BA are known to prevent the accumulation of amyloid β peptides in Alzheimer’s disease; protect against mitochondrial damage in Parkinson’s disease; protect against apoptosis, mitochondrial damage, and ubiquitin accumulation in Huntington’s disease; and protect against apoptosis in amyotrophic lateral sclerosis.

Twenty different BA and their receptors have been identified in the brain, implying that BA have physiological and pathophysiological roles in this tissue (Figure 4), and indeed, many studies of their physiological roles in the brain have been reported.

### 5.1. The Role of BA in the Brain

BA affect the functioning of the receptors for several neurotransmitters, including M_2_ and M_3_ muscarinic acetylcholine receptors, γ-aminobutyric acid (GABA) type A (GABA_A_) receptors, and N-methyl-D-aspartate (NMDA) receptors. TCA, GDCA, and TDCA activate the M_2_ receptor, while DCA activates the M_3_ receptor. M_2_ receptors are distributed throughout the brain and are crucial for cognitive function. However, M_3_ receptors are localized to neurons, which project to regions such as the hippocampus and substantia nigra, although they are also expressed at low levels throughout the brain. Thus, BA may affect cognitive function, memory, and learning [140]. NMDA receptors are ionotropic glutamate receptors that are activated by the simultaneous binding of both glutamate and D-serine or glycine [141,142]. Their activation causes Ca^2+^ influx, which can induce long-term potentiation and long-term depression. Therefore, appropriate NMDA receptor activation is important for learning and memory [143]. In contrast, the GABA_A_ receptor is an ionotropic GABA receptor that is a ligand-gated chloride ion channel. The activation of GABA_A_ receptor causes an influx of chloride ions, leading to the hyperpolarization of neurons and the inhibition of neurotransmission [144]. CDCA, DCA, and CA block both GABA_A_ and NMDA receptors [145]. Histaminergic neurons in the tuberomammillary nucleus (TMN) of the hypothalamus play an important role in arousal and wakefulness [146] and express GABA_A_ receptors; thus, the suppression of histaminergic neuronal activation in the TMN by GABA induces sleep [147]. Conversely, UDCA increases arousal by blocking GABA_A_ receptors on TMN neurons [148]. Moreover, a recent study has demonstrated that TUDCA can induce neurogenesis in adult rats [149]. Adult neurogenesis is the process of generating new functional neurons that are added to the adult brain and occurs in two specific regions: The subgranular zone (SGZ) of the dentate gyrus (DG) in the hippocampus and the subventricular zone (SVZ), located in the walls of the lateral ventricles. Neural stem cells (NSCs) in the SGZ of adult mammals generate neurons in the DG. NSCs in adult human SVZ may produce functional neurons in the striatum by migrating there. In contrast, NSCs in adult rodent SVZ may produce functional neurons in the olfactory bulb [150]. In the rat, TUDCA increases the proliferation and neural differentiation of NSCs in the SVZ, but not in the DG [149].

### 5.2. The Role of BA in Neurodegenerative Diseases

Alzheimer’s disease is characterized by memory loss, dementia, and morphological changes in the brain and is a common progressive neurodegenerative disease. The main pathological feature of the brains of patients with Alzheimer’s disease is the accumulation of amyloid β peptides and tangles of tau protein [151]. The processing of amyloid precursor protein (APP) by β and γ secretases generates amyloid β peptide [152,153] and the γ secretase complex includes presenilin 1 (PS1), which is associated with the maturation of V-ATPase, responsible for the acidification of lysosomes. PS1 dysfunction, thus, leads to an impairment in lysosomal acidification and function [154,155]. The accumulation of amyloid β peptides is relevant to the dysfunction of both APP and γ secretase, and APP/PS1 double knockout mice, which express both mutated human APP and PS1, are used as a model of Alzheimer’s disease. Interestingly, TUDCA reduces the accumulation of amyloid β peptides in the hippocampus and frontal cortex and rescues memory deficits in APP/PS1 double knockout mice [156,157]. In humans, plasma CA concentrations in patients with Alzheimer’s disease are significantly lower than those in control subjects, and the TCA concentration in the brain of patients with Alzheimer’s disease is also significantly lower [42]. In contrast, the plasma concentration of LCA, a secondary BA, is significantly higher in patients with Alzheimer’s disease than in controls [158]. Furthermore, it has been shown that the ratio of DCA (secondary BA) to CA (primary BA) in serum is significantly higher in Alzheimer’s patients [159]. These studies indicate the relationship between BA and Alzheimer’s disease, and the importance of the brain-gut-microbiome axis. However, morphological and functional abnormalities in mitochondria have also been identified in patients with Alzheimer’s disease [160,161]. In fibroblasts from Alzheimer’s disease patients, the mitochondrial membrane potential (MMP) is lower, and there is mitochondrial elongation. Dynamin-related protein 1 (DRP1) plays an important role in mitochondrial fission and is essential for mitochondrial quality control [162]. Although the expression of DRP1 is lower in fibroblasts from Alzheimer’s disease patients, UDCA increases its expression [163].

Parkinson’s disease is another common progressive neurodegenerative disease that is characterized by tremors, muscle stiffness, loss or impairment of voluntary movements, slowness of movement, and postural instability [164]. Mutations in phosphatase and tensin homolog-induced putative kinase 1 (PINK1) and parkin are found in early-onset Parkinson’s disease and are important in the progression of Parkinsonism [165,166,167,168]. PINK1 and parkin initiate mitophagy and nuclear dot protein 52 kDa (NDP52), and optineurin are essential for this process [169,170]. 1-Methyl-4-phenyl-1,2,3,6-tetrahydropyridine (MPTP) and rotenone treatment have been widely used in the creation of a model of Parkinson’s disease. Both toxins inhibit complex I in mitochondria, leading to neuronal mitochondrial damage and Parkinsonism [171,172]. However, TUDCA protects neurons from MPTP-induced oxidative stress and neurotoxicity in the midbrain and striatum of mice [173]. TUDCA induces nuclear factor erythroid 2-related factor 2 (Nrf2) expression and reduces the generation of reactive oxygen species, and indeed, this anti-oxidant effect of TUDCA may be Nrf2-dependent [174]. Moreover, TUDCA ameliorates clinical signs induced by MPTP, such as the increase in swimming latency, foot-dragging, and tremors. In addition, TUDCA inhibits the loss of dopaminergic neurons and reduces the loss of MMP and mitochondrial mass elicited by MPTP [175]. Furthermore, TUDCA increases the expression of PINK1, parkin, and the ratio of LC3-II/lC3-I, implying that TUDCA induces mitophagy [176]. In addition, in the rotenone-induced rat model of Parkinson’s disease, UDCA ameliorates abnormalities in the mitochondria of striatal neurons, such as irregular swelling and loss of cristae [177].

Huntington’s disease is an autosomal dominant inherited neurodegenerative disease caused by a CAG trinucleotide expansion encoding polyglutamine (polyQ) at the N-terminus of Huntingtin (HTT). Huntington’s disease is characterized by motor dysfunction, cognitive decline, and psychiatric disturbances [178,179]. Chemical and genetic models of Huntington’s disease have been studied [180,181]. Firstly, 3-nitropropionic acid (3-NP) is an inhibitor of succinate dehydrogenase (complex II) in mitochondria and induces degeneration of the caudate-putamen, which is also present in Huntington’s disease [180]. 3-NP treatment is associated with swelling of striatal mitochondria, abnormal mitochondrial membrane structure, and apoptosis. However, TUDCA prevents mitochondrial damage and apoptosis and ameliorates the sensorimotor deficits induced by 3-NP [182]. Secondly, R6/2 transgenic mice possess a genomic fragment containing exon 1 of the human *Huntingtin* gene, which carries 144 CAG repeats, and are the most widely used model of Huntington’s disease [181,183]. In these mice, TUDCA prevents the striatal apoptosis and cerebral and striatal atrophy [184]. The accumulation of HTT and ubiquitin are also symptoms of Huntington’s disease, and ubiquitin is recruited to polyglutamine-expanded HTT fragments [185]. TUDCA reduces the accumulation of ubiquitin in the striatum of R6/2 transgenic mice and ameliorates their sensorimotor deficits [184].

Amyotrophic lateral sclerosis (ALS) is a progressive and ultimately fatal disease that is characterized by the degeneration of both upper and lower motor neurons, leading to muscle weakness, atrophy, and paralysis [186]. Impairments in superoxide dismutase 1 (SOD1), C9ORF72, TAR DNA-binding protein of 43 kDa (TDP-43), and fused in sarcoma (FUS) are molecular features of this disease [187]. Transgenic mice that carry the glycine 93 to alanine mutation of human SOD1 (hSOD1^G93A^) are the most studied model of ALS [188]. NSC-34 cells carrying hSOD1^G93A^ also represent a useful model of ALS-affected motor neurons [189]. Glycoursodeoxycholic acid (GUDCA) prevents the apoptosis of NSC-34 cells carrying hSOD1^G93A^ [190], and clinical trials of the use of TUDCA in patients with ALS have shown an improvement in muscle function and survival time, without adverse effects [191].

## 6. Concluding Remarks

The existence of 20 BA in the brain has been demonstrated. These 20 BA include uncojugated BA (CA, CDCA, DCA, and UDCA) and conjugated BA (GDCA, TCA, TDCA, and TUDCA), which are related to the brain physiology and/or to pathophysiology [6]. In addition, receptors and ion channels that are activated by BA are also expressed in this tissue. These findings imply that BA have a neurological function. The effects of several BA to protect against neurodegeneration are associated with inhibition of the accumulation of amyloid β peptides, mitochondrial damage, and apoptosis. However, BA have been shown to activate many receptors and ion channels, through which they may also play a neuroprotective role. Especially, TUDCA is the bile acid with the most diversity of actions on the human organism. Although the mechanism whereby TUDCA might induce adult neurogenesis remains unclear, it is possible that other BA also affect neurogenesis. Neuroprotective effects and the induction of adult neurogenesis represent important approaches to the therapy of neurogenerative diseases and brain injury. Both conjugated and unconjugated BA can enter the brain by crossing the BBB or using transporters. Moreover, UDCA, which is approved by the US Food and Drug Administration for the treatment of primary biliary cirrhosis [192], can enter the cerebrospinal fluid after oral administration [193]. Thus, the oral administration of BA may represent a feasible approach for the treatment of neurodegenerative diseases and brain injury in the future.

Elucidation of the detailed mechanisms involved in the synthesis, signaling, and physiological functions of BA in the brain should help the development of novel therapeutic and diagnostic strategies for brain diseases.
